# Whole-genome sequencing reveals cellular origin of concomitant chronic lymphocytic leukemia and multiple myeloma: a case report

**DOI:** 10.1080/15384047.2024.2403203

**Published:** 2024-09-18

**Authors:** Jianing Zhang, Ji Ma, Ying Li, Xiao Lv, Lili Feng

**Affiliations:** a Department of Radiation Oncology, Shandong Cancer Hospital and Institute, Shandong First Medical University and Shandong Academy of Medical Sciences, Jinan, China; b Department of Hematology, Shandong Cancer Hospital and Institute, Shandong First Medical University and Shandong Academy of Medical Sciences, Jinan, China; c Department of Hematology, Shandong Provincial Hospital Affiliated to Shandong First Medical University, Jinan, China

**Keywords:** Chronic lymphocytic leukemia, multiple myeloma, whole-genome sequencing, cellular origin, case report

## Abstract

Chronic lymphocytic leukemia (CLL) and multiple myeloma (MM) are hematological disorders affecting B cells. The clonal relationship between CLL and MM has not always been clarified, although this information is critical to understanding its pathogenesis. Here, we present a rare clinical case of synchronous CLL and MM. Whole-genome sequencing (WGS) was performed using malignant lymph node (LN) and bone marrow (BM) tissues. Based on the high consistency of single nucleotide variants (SNVs), significantly mutated genes (SMGs), copy number variations (CNVs), different B cell receptor (BCR) IGH rearrangement features in LN and BM, and the different light-chain expression patterns in CLL and MM cells, we concluded that CLL and MM cells from this patient originated from the same hematopoietic stem cell/progenitors, different pro-B cells and suffered oncogenic mutations at different B cell differentiation stages. Depth analysis of genome features using WGS provides a new method to explore the process of malignant B cell genesis.

## Background

Chronic lymphocytic leukemia (CLL) is the most common adult leukemia in Western countries and is characterized by the clonal expansion of CD5^+^CD23^+^ B cells in the blood, bone marrow (BM), and secondary lymphoid tissues.^1^ Multiple myeloma (MM) is the second most common hematological malignancy and is characterized by the presence of abnormal clonal plasma cells in the BM.^2^ CLL and MM are hematological disorders that occur at different stages of B-cell development. Recent studies have provided evidence that the earliest genetic and epigenetic alterations eventually leading to CLL may occur in pluripotent hematopoietic stem cells (HSCs).^3^ MM is the hematological malignancy of plasma cells, which represents the final differentiation stage of B cells.

The precise cellular origin of CLL and MM remains debatable. Maturation of a normal B-cell precursor to a mature plasma cell involves B-cell receptor (BCR) rearrangement and somatic mutation of the immunoglobulin variable (V) region genes. These events occur at distinct stages of development, and when a B cell undergoes neoplastic transformation, the genetic imprint reflects the clonal history of the cell of origin.^4^

The concomitant occurrence of CLL and MM in a patient provides a unique opportunity to study the cells of origin for CLL and MM. However, this case is extremely rare.^5^ Here we present a patient with synchronous CLL and MM. We performed whole-genome sequencing (WGS) with malignant lymph node (LN) and BM tissues and analyzed the genomic features to explore the cellular origin of CLL and MM.

## Case presentation

**Figure 1.**
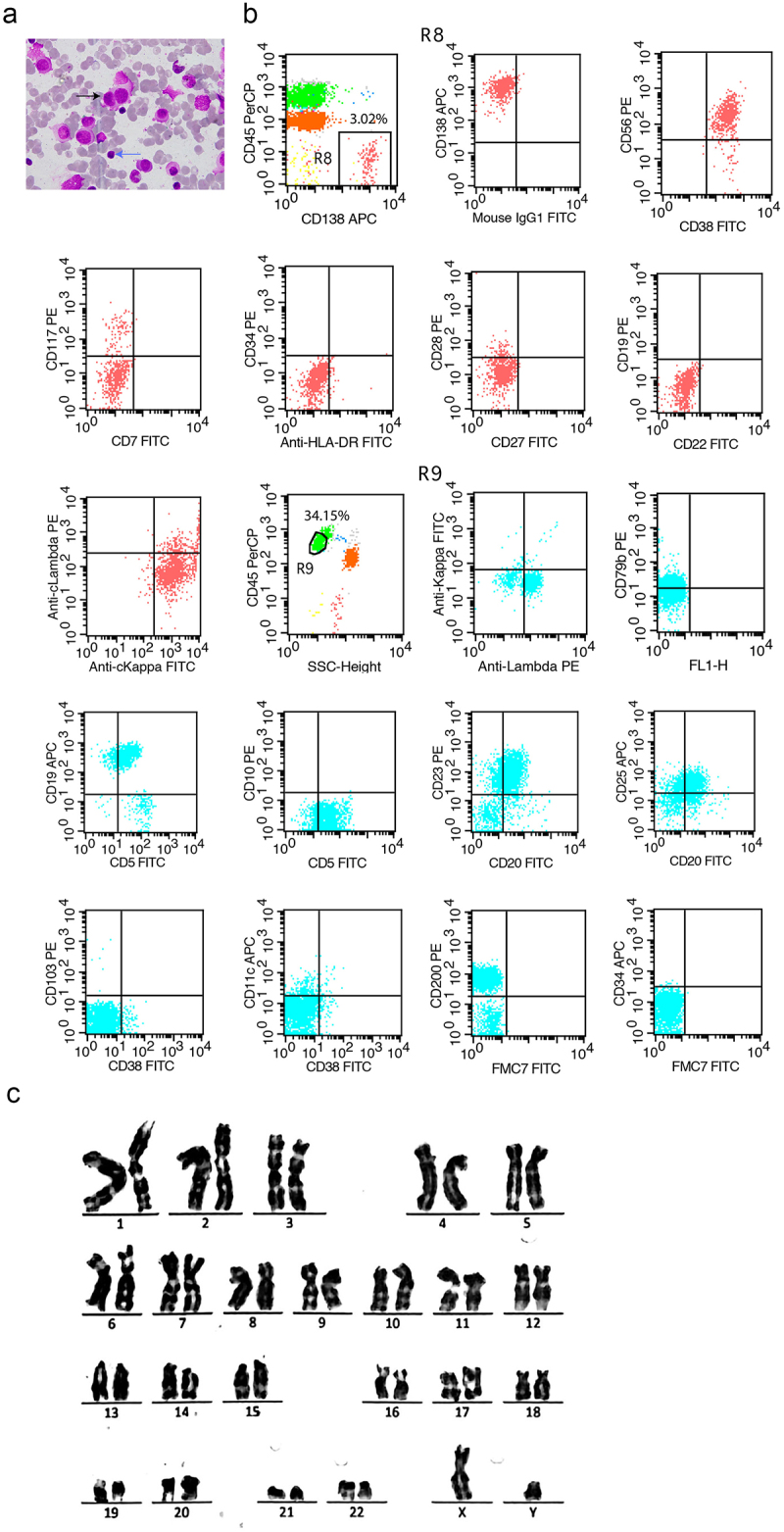
BM cells morphology, flow cytometric analysis and chromosomal analysis results for this patient. (a) Representative images of BM cells showing MM cell (black arrow) and mature lymphocyte (blue arrow). (b) Monoclonal CLL cells and MM cells were analyzed with flow cytometry. R8 showed the population of MM cells and R9 showed the population of CLL cells. (c) Chromosomal analysis results for this patient.

A 61-year-old man was referred to the Hematology Department because of backache. Laboratory screening revealed increased lymphocytes (7.11 × 10^9^/L, normal reference value 1.1–3.2 × 10^9^/L) and monoclonal free κ chain (3550 mg/L, normal reference value 3.30–19.4 mg/L) in blood in the absence of anemia, thrombocytopenia and kidney failure. The level of free κ chain in urine also increased significantly (>4075 mg/L, normal reference value 0.39–15.1 mg/L). The levels of albumin and β_2_-microglobulin in serum were 31.3 g/L and 4585.6 ng/ml, respectively. Magnetic resonance imaging (MRI) revealed multiple osteolytic lesions in the skull and lumbar vertebrae. Ultrasound examination revealed multiple enlarged LN in the neck, armpit, and groin, in the absence of hepatomegaly and splenomegaly. A BM smear showed 26% atypical plasma cells and 12% mature lymphocytes (normal reference value 15.7–29.8%) (Figure 1a). Flow cytometric analysis confirmed that the mature lymphocytes were monoclonal cells expressing CLL markers (CD19^+^, CD5^+^, CD23^+^,CD25^+^, CD20^+^, CD200^+^, cLambda^+^, CD79b^dim^, CD10^−^, FCM7^−^,CD103^−^, CD38^−^, CD11c^−^, CD34^−^, and cKappa^−^, Figure 1b, R9, 34.15%), whereas monoclonal plasma cells were CD38^+^, CD138^+^, CD56^+^, cKappa^+^, CD117^−^, CD19^−^, CD7^−^, CD27^−^, CD28^−^, CD34^−^, HLA-DR^−^, CD22^−^, CD45^−^, and cLambda^−^ (Figure 1b, R8, 3.02%). These results provided evidence to support the presence of separate clones in BM. Chromosomal analysis revealed a normal karyotype (46, XY [20], Figure 1c). These results supported the diagnosis of CLL (Binet stage B, Rai stage I) and MM (Durie – Salmon stage III, International Stage System stage II).

To explore the cellular origin of these malignant cells, genomic DNA was extracted from paraffin-embedded BM and LN tissues using the QIAamp DNA FFPE Tissue Kit (Qiagen, Germany), according to the manufacturer’s protocol. DNA from peripheral blood mononuclear cells (PBMCs) was extracted with DNeasy Blood & Tissue Kits (Qiagen, Germany) according to the manufacturer’s instructions. The qualified DNA library was sequenced using the Illumina HiSeq X Ten. Paraffinized BM and malignant LN tissues were confirmed by two pathologists independently. CLL cells accounted for 93% of the LN cells. The percentage of MM and CLL cells in the BM was 30% and 20%, respectively.

**Figure 2.**
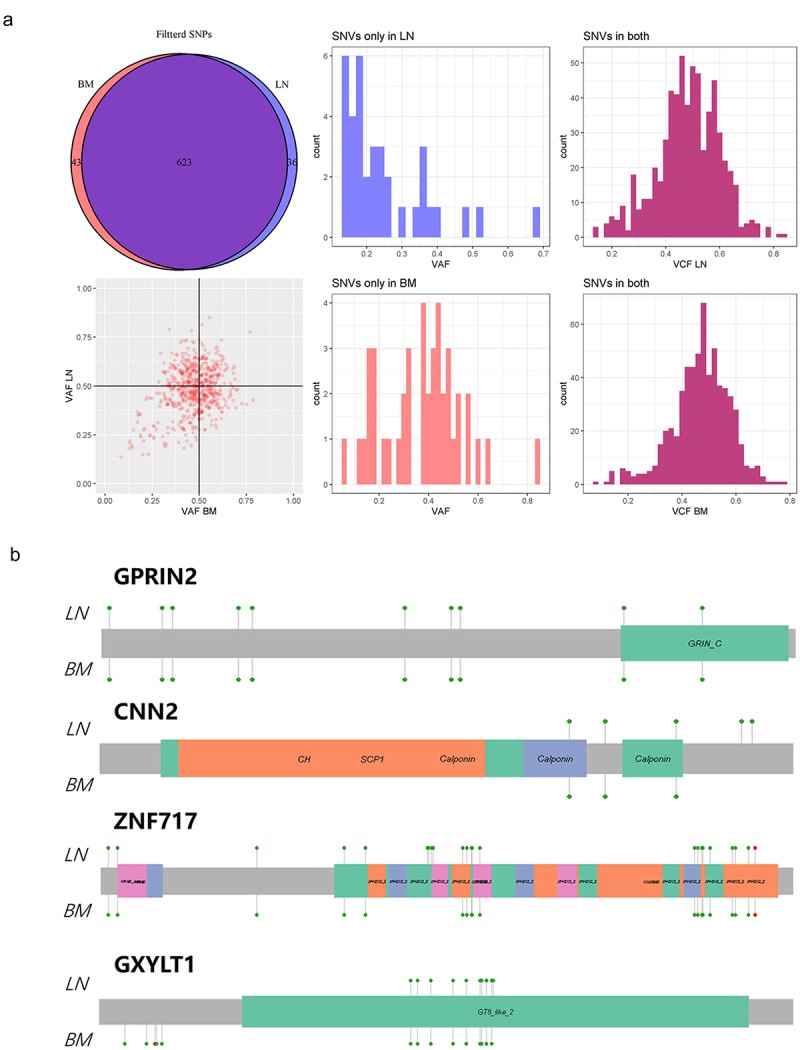
Tumor-related SNVs in LN and BM. (a) SNVs were shown in the venn. (b) *GPRIN2, CNN2, ZNF717* and *GXYLT1* gene were selected for visualization.

Tumor-related Single nucleotide variants (SNVs) were analyzed and shown in Venn (Figure 2a). We found that SNV events were highly consistent between the LN and BM genomes. After excluding germline mutations, the number of SNVs of LN and BM were 659 and 666, respectively. For the majority of common mutations in LN and BM (*n* = 623), the mutation frequency was approximately 0.5 (Figure 2a). These were also likely germline mutations. We selected some genes with more mutated nucleotides for visualization (*GPRIN2*, *GXYLT1*, *ZNF717* and *CNN2*, Figure 2b). We found that most mutation sites in LN were the same as those in BM (such as *GPRIN2*), although there were differences in some genes (*GXYLT1*, *ZNF717* and *CNN2*). Only a small number of special SNVs were detected in the LN and BM genomes, suggesting that SNVs may not be driving factors for hematological malignancies.

Significantly mutated genes (SMGs) were defined as those genes where SNVs or insertions/deletions occur at a significantly higher rate compared to the background mutation rate. The identification of SMGs was called with the MuSic software, which employed all somatic mutations from tumor samples as background, to conduct statistical tests on various mutation types across genes. The results for the SMGs were shown in Table 1. KEGG pathway enrichment analysis of SMGs was carried out using PathScan (a MuSic tool), and the results were shown in Table 2. We found that SMGs and enriched KEGG pathways were consistent in the two samples, demonstrating the high consistency of SMGs in the LN and BM genomes.

**Table 1.** The statistical results of SMGs.

Gene	Indels	SNVs	Tot Muts	Sample Affect	P-value CT	FDR CT
*AHNAK2*	0	60	60	2	0	0
*ALPK2*	2	14	16	2	0	0
*MUC16*	0	41	41	2	0	0
*KIR2DL3*	0	15	15	2	2.22882925956578e-24	6.102e-21
*DNAH14*	2	18	20	2	7.02177027673671e-22	1.682e-18
*ZNF717*	0	13	13	2	1.02401982228697e-21	2.180e-18
*CYP2A7*	0	12	12	2	6.45188442850414e-21	1.236e-17

**Table 2.** KEGG pathway enrichment analysis of SMGs.

Pathway	Samples affected	Total Variations	p-value
Inflammatory mediator regulation of TRP channels	2	31	1.426e-31
Antigen processing and presentation	2	30	2.753e-31
Arachidonic acid metabolism	2	24	4.623e-30
Herpes simplex infection	2	33	5.997e-30
Phosphatidylinositol signaling system	2	32	7.003e-30
Retinol metabolism	2	20	7.817e-30
Calcium signaling pathway	2	32	1.207e-29

**Figure 3.**
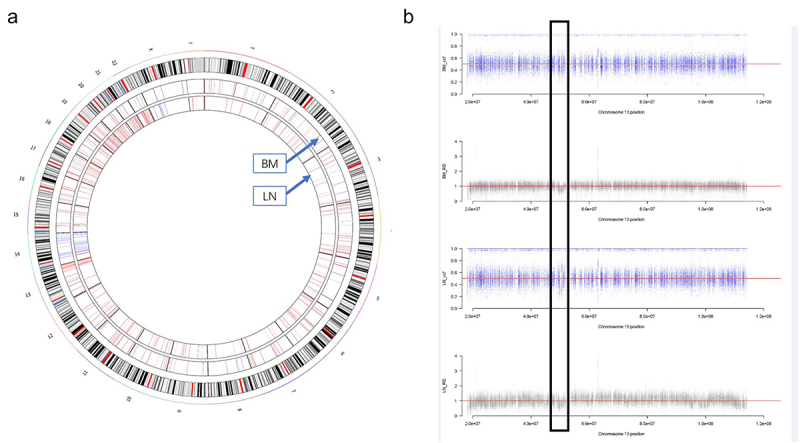
CNVs was displayed with circos plot and segments of chromosome 14 were selected to visualize. (a) CNVs in all chromosomes were shown with circus plot. Red lines represented amplification and blue lines represented the reduction of CNVs. (b) Chromosome 14 was visualized to show the reduction of CNVs in LN and BM samples.

Copy number variations (CNVs) were analyzed with CNVnator, and the results were displayed with a circos plot (Figure 3a). CNVs analysis showed a heavy burden of copy number events in the whole genome, which might be the driving factors for CLL and MM. Although the copy number events involved more regions in LN (totally 24.51 Mb genome-wide) than in BM (3.99 Mb in totally), the aberrant patterns were similar within these two samples, such as the amplification in chromosome 13q and the deletion in chromosome 14. This consistency was further documented in a painstaking comparison of each aberrant segment. Chromosome 14 was selected to visualize the distribution of the coverage depth and mutation frequency (Figure 3b). Both BM and LN samples showed a reduction in CNVs, especially in LN (black frame), which was the result of a high proportion of malignant B cells.

**Figure 4.**
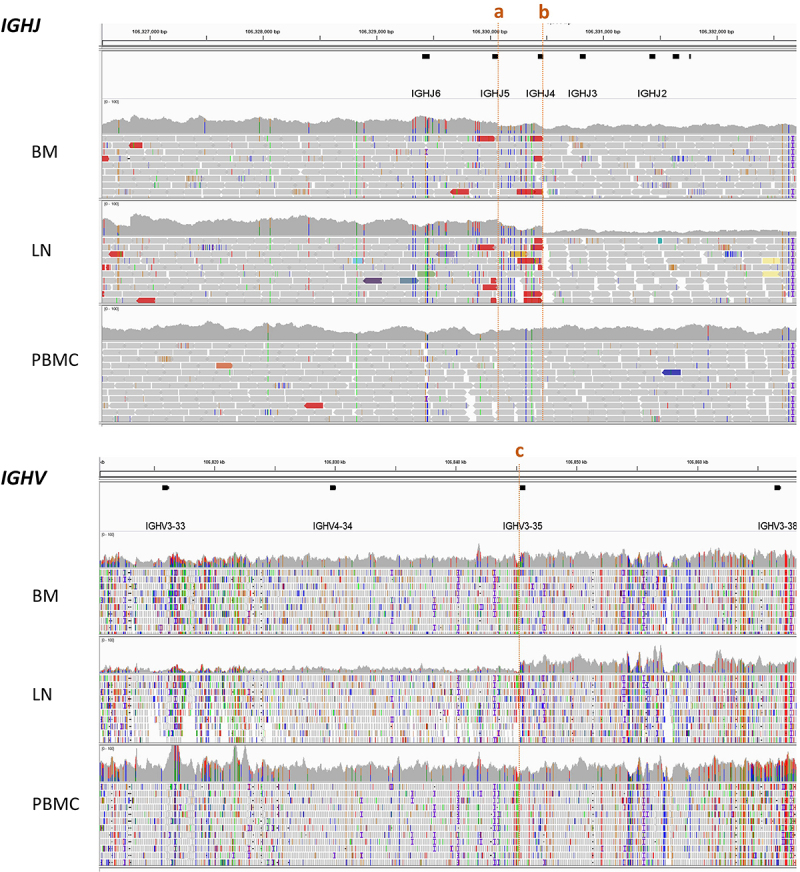
WGS sequencing depth display in BCR IGHJ and IGHV rearrangement in LN and BM.

During the development from pluripotent hematopoietic stem cells to mature B cells, the cells undergo heavy chain gene (IGH) rearrangement and light chain gene (IGK, IGL) rearrangement, resulting in cell-specific BCR, which is strong evidence for the clonal proliferation of B cells. To further explore the clonality in our case of CLL with MM, we analyzed the IGH rearrangement patterns in the LN and BM. The sequencing depth of the PBMCs genome from a healthy volunteer was analyzed using the same method to show the mappability of this region (Figure 4). In LN, the average sequencing depth was 47 and dropped to 18 within the region from IGHJ4 (b) to IGHV3–35 (c). This reduction was more obvious when compared with the adjacent regions (average sequencing depth 64). The reason for the sequencing depth reduction between IGHJ4 and IGHV3–35 might be DNA shearing, which indicated IHG rearrangement at IGHJ4 and IGHV3–35. This suggested that the monoclonal B cells in LN samples were in the stage after IGH rearrangement (after pro-B). However, the rearrangement features of IGH in the BM samples were not as obvious as those in LN. The average sequencing depth in the BM was 51, whereas there was no obvious decrease within the IGH region. The rearrangement in JH might occur at IGHJ5 (a), but the rearrangement location in the VH was uncertain. The different rearrangement features of IGH in LN and BM indicated that CLL and MM cells originated from different pro-B cells.

## Discussion

The occurrence of MM and a second B-cell neoplasm in the same patient is rare. In our study, we performed WGS for a patient with synchronous MM and CLL to analyze genomic features and explore the clonal origins of these malignant cells. Based on the high consistency of SNVs, SMGs, and CNVs, the different BCR IGH rearrangement features in LN and BM, and the different light chain expression in CLL and MM cells, we concluded that CLL and MM cells originated from the same hematopoietic stem cell/progenitors, different pro-B cells, and suffered oncogenic mutations at different B cell differentiation stages.

To explore the origin of malignant cells in this patient, we first analyzed genomic SNVs, SMGs, and CNVs features in the LN and BM. To obtain accurate analysis results without normal tissues from this patient as a control, we strictly filtered germline mutations and acquired tumor-related SNVs. We found that the SNVs, SMGs, and CNVs features in LN and BM were highly consistent, suggesting that CLL and MM cells originate from the same HSCs/progenitors. CNVs events were extremely common and were possible driving factors for these two hematological malignancies.

B cell development originates from HSCs, which can successively differentiate into multipotent progenitors, common lymphoid progenitors (CLP), pre-pro-B cells, pro-B cells, pre-B cells, naive B cells, transitional B cells, and eventually mature B cells. At the molecular level, B cell ontogeny commences with the IGH rearrangement (pro-B), which contains four major domains: the variability domain (VH), diversity domain (DH), joining domain (JH), and constant domain. If the rearrangements are in frame, or “productive,” the pro-B cell will then rearrange the IGL (pre-B) with first attempts to rearrange the IGLκ gene. If productive, mature B cells will be able to produce IgMκ, which is expressed on the surface of B-cell. If unsuccessful, the B cell will then rearrange the IGLλ gene, leading to the production of IgMλ.^6^ Mature naïve B cells can then transition from BM into secondary lymphoid tissues, where they continue their differentiation. This part of differentiation is T-cell-independent (TI) or T-cell-dependent (TD). Compared with TI, TD cytokine stimulation induces more complex B-cell activation in the germinal center (GC), which involves somatic hypermutation (SHM) and class-switch recombination (CSR).^6^ The mature B cells then express a different Ig, either IgG, IgA, or IgE.

**Figure 5.**
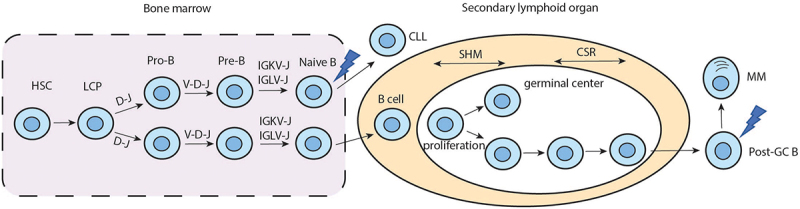
The schematic diagram of cellular origin of malignant cells from this patient.

As BCR rearrangement features were strong evidence for the clonal proliferation of B cells, we analyzed the rearrangement features of IGH. We found that the sequencing depth reduced significantly from IGHJ4 to IGHV3–35 in LN, which might be caused by DNA shear between them. For MM cells, the rearrangement location in the BM may be IGHJ5, and it was uncertain in VH. The different rearrangement features of IGH in LN and BM indicated that CLL and MM cells derived from different pro-B cells (Figure 5).

We then analyzed the developmental stage of B cells suffering the carcinogenic genetic hits in the malignant cells. IgHV-M and IgHV-UM CLL cells have different cells of origin.^7^ It is indicated that IgHV-M CLLs derives from post-GC CD5^+^CD27^+^ B cells. However, whether IgHV-UM CLLs are derived from pre-GC (naive) B cells or GC-independent antigen-experienced B cells remains unclear.^8^ Our FISH analysis revealed that CLL cells from this patient were IgHV-UM cells. Moreover, our phenotyping data revealed that CLL B cells expressed λ light chains. Given the above results, we concluded that CLL cells probably suffered carcinogenic genetic hits in the naive B cells stage.

Mounting evidence supports the hypothesis that oncogenic transformation in MM occurs within secondary lymphoid organs, similar to IGHV-M CLL cells.^6^ It is also suggested that almost all myelomas are initiated by mutations associated with TD responses. The fact that more than 90% of myelomas expressed class-switched IgH implicated that myeloma-initiating alterations were a result of errors in CSR.^9^ Laboratory tests showed that MM cells from this patient expressed the κ chain and secreted high amounts of monoclonal free κ chain. Based on the secretion of the κ chain and IgH rearrangement features, we concluded that the oncogenic transformation of MM cells occurred in post-GC B cells, where B cells had undergone IGH and IGL rearrangement, SHM, and CSR (Figure 5). However, the absence of immunoglobulin heavy chain production under these conditions has not yet been fully elucidated. Several mechanisms have been proposed to explain the absence of heavy chain production, including abnormalities at the genomic, transcriptional, or translational level; instability of mRNA; or rapid degradation of the protein.^10^

Our study had some limitations. First, the unavailability of fresh samples made it impossible to obtain purified CLL and MM cells. Second, the absence of normal tissue as a control challenges the analysis of the WGS data. To obtain accurate results without normal control tissue from this patient, we filtered the following data: (1) mutations in the exonic region; (2) mutation frequency (VCF) less than 0.95; (3) non-synonymous mutation; (4) harmful mutations in at least one function prediction score (FATHMM, LRT, PolyPhen-2, MutationTaster, SIFT, RadialSVM, and CADD) or annotated in the cosmic database; and (5) the frequency of the mutation was less than 0.05 in normal population. Furthermore, our present data cannot prove the existence of premalignant HSCs/progenitors, and our conclusion was drawn not only from our data but also from some previously published hypotheses. However, the synchronous presence of CLL and MM in the same patient is extremely rare, and this provides vital help for the identification of the cellular origin of CLL and MM. Although WGS is not the standard analysis method for BCR rearrangement, it provides a new method for analyzing the features of BCR rearrangement.

## Conclusions

In summary, we described a patient with CLL and MM. Genomic and phenotyping features of CLL cells and MM cells indicated that CLL and MM cells originated from the same hematopoietic stem cell/progenitors, different pro-B cells, and oncogenic mutations occurred independently at different B cell differentiation stages. The described clinical case helped us explore the cellular origin of CLL and MM. Depth analysis of genome features using WGS provides a new method for exploring the process of malignant B cell genesis.

## Abbreviations

CLL, chronic lymphocytic leukemia; MM, multiple myeloma; WGS, whole-genome sequencing; LN, lymph node; BM, bone marrow; SNVs, single nucleotide variants; SMGs, significantly mutated genes; CNVs, copy number variations; BCR, B cell receptor; HSCs, hematopoietic stem cells; IgHV, immunoglobulin heavy chain gene; CLP, common lymphoid progenitors; TI, T-cell independent; TD, T-cell dependent; GC, germinal center; SHM, somatic hypermutation; CSR, class-switch recombination.
